# The influence of early maladaptive schemas on psychological flexibility of college students: a moderated mediation model

**DOI:** 10.3389/fpsyg.2026.1751416

**Published:** 2026-07-07

**Authors:** Sicen Zhang, Quandong Liu, Qiuying Zhang, Menglu Jia, Yi Song, Lixia Zhang

**Affiliations:** 1 School of Public Health, Shaanxi University of Chinese Medicine, Xianyang, China; 2 Hainan General Hospital, Haikou, China; 3 The Second Affiliated Hospital of Zhejiang University School of Medicine Lanxi Hospital, Lanxi, China; 4 Beijing Huilongguan Hospital, Beijing, China

**Keywords:** college students, early maladaptive schemas, perceived social support, psychological flexibility, self-compassion

## Abstract

**Background:**

This study aimed to examine the associations between early maladaptive schemas (EMSs) and psychological flexibility among college students, as well as the mediating role of self-compassion and the moderating role of perceived social support.

**Methods:**

A total of 1,184 college students were surveyed using the short form of the Young Schema Questionnaire, the short form of the Avoidance and Fusion Questionnaire, Neff’s Self-Compassion Scale, and the Perceived Social Support Scale.

**Results:**

EMSs were negatively correlated with self-compassion and perceived social support, and positively correlated with psychological inflexibility. Perceived social support was positively correlated with self-compassion and negatively correlated with psychological inflexibility, while self-compassion was negatively correlated with psychological inflexibility. Structural equation modeling indicated that EMSs were negatively associated with self-compassion, which in turn was negatively associated with psychological inflexibility. EMSs also showed a significant positive direct association with psychological inflexibility. In addition, perceived social support moderated the association between EMSs and self-compassion.

**Conclusion:**

EMSs showed significant direct and indirect associations with psychological flexibility. Specifically, self-compassion partially mediates the relationship between EMSs and psychological flexibility, while perceived social support moderates the pathway between EMSs and self-compassion. These findings provide empirical support for improving college students’ mental health and developing targeted psychological interventions.

## 1 Introduction

In the rapidly changing modern society, college students encounter a variety of demands and obstacles, including academic competition, career planning, and emotional management. Understanding how to maintain psychological wellbeing, deal with adversity, and achieve self-development in such a setting has become a critical concern for both academia and society. Psychological flexibility refers to an individual’s ability to change their feelings and behaviors in reaction to stress, hardship, and ambiguity. This concept originates from the Acceptance and Commitment Therapy (ACT) framework and consists of six core processes (Harris, 2019) including acceptance, cognitive diffusion, being present, self-as-context, values, and committed action. Psychological flexibility is a crucial factor influencing college students’ mental health (Imani et al., 2017). Students with high psychological flexibility are better equipped to deal with life’s uncertainties and obstacles, which benefits both personal development and social involvement. In contrast, psychological inflexibility limits students’ adaptability in learning, emotional regulation, interpersonal interactions, and career planning, ultimately affecting their overall development and mental wellbeing. Therefore, investigating the factors that influence college students’ psychological flexibility can help enhance psychological health and improve their ability to cope with stress and challenges.

Early maladaptive schemas (EMSs) are enduring cognitive–emotional patterns that originate in childhood or adolescence and persist into adulthood, shaping individuals’ emotions, cognition, and behavior. These maladaptive schemas are typically rooted in unmet emotional needs, adverse early experiences, or dysfunctional family interactions (Young et al., 2006). Young identified 18 types of EMSs, which are organized into five domains: disconnection and rejection, impaired autonomy and performance, impaired limits, other-directedness, and over vigilance and inhibition (Zhang and Zhang, 2026). Emerging evidence has begun to link EMSs with psychological flexibility, indicating that maladaptive schemas may be associated with diminished psychological flexibility (Zhang et al., 2025). Conceptually, EMSs are characterized by negative self-representations, cognitive rigidity, and experiential avoidance, all of which have been identified as central impediments to psychological flexibility (Zhang et al., 2026). From the perspective of ACT, psychological flexibility is defined as the capacity to remain open to internal experiences while engaging in value-directed behavior. In contrast, EMSs may reinforce cognitive fusion and avoidance tendencies, thereby undermining this capacity (Özdemir, 2021). Despite accumulating evidence supporting an association between EMSs and psychological flexibility, the mechanisms underlying this relationship remain insufficiently understood. In particular, the extent to which this association may operate through internal psychological processes such as self-compassion warrants further empirical investigation.

Self-compassion refers to an individual’s ability to respond to personal suffering with understanding, kindness, and non-judgment. It is made up of three fundamental components: self-kindness, common humanity, and mindfulness (Gong et al., 2014). The ACT framework emphasizes that psychological flexibility is rooted in acceptance and awareness (Harris, 2019), which is consistent with the primary idea of self-compassion—treating oneself non-judgmentally. Therefore, self-compassion helps individuals reduce negative emotions, enhancing psychological flexibility and preventing emotional entanglement. Studies have confirmed a positive correlation between psychological flexibility and self-compassion (Pyszkowska and Rönnlund, 2021). Additionally, research has found a negative correlation between EMSs and mindfulness (Flink et al., 2018) a key component of self-compassion. Fischer found that EMSs promote cognitive fusion and experience avoidance, both of which reduce psychological flexibility (Fischer et al., 2016).

Social support refers to the emotional, informational, and instrumental resources individuals receive from their families, friends, and social networks when facing stress. Unlike objective social support, perceived social support represents people’s subjective evaluations of the social support they receive (Cohen and Wills, 1985), which is important for stress alleviation, emotional regulation, and mental health improvement (Lakey and Orehek, 2011). Research suggests that perceived social support can buffer the negative effects of EMSs, providing individuals with opportunities to correct and mitigate the influence of maladaptive schemas, thereby enhancing emotional regulation abilities (Shahbeik et al., 2023). Furthermore, perceived social support influences self-compassion (Wilson et al., 2020). The conservation of resources theory states that gaining and keeping resources helps people cope with adversity while also reducing stress and bad emotions. These resources encompass not just external material help, but also social and emotional support, time, and psychological resilience. Perceived social support, a psychological resource, and self-compassion, a resource-protection mechanism, work together to promote mental health (Maheux and Price, 2016).

**Figure 1:**
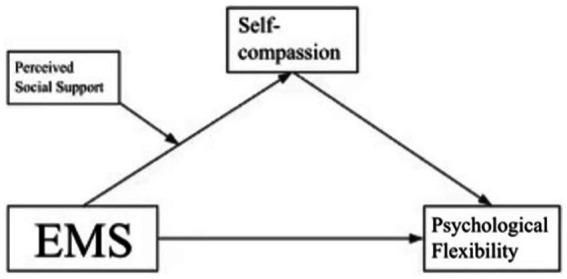
The hypothesized moderated mediation model.

In summary, the present study constructs a moderated mediation model to examine the association between EMSs and psychological flexibility among college students, with self-compassion serving as a mediator and perceived social support as a moderator. The proposed model is shown in Figure 1. Based on the theoretical framework outlined above, the following hypotheses are proposed:

*H1*: EMSs negatively predict psychological flexibility.

*H2*: Self-compassion mediates the relationship between EMSs and psychological flexibility.

*H3*: Perceived social support moderates the relationship between EMSs and self-compassion.

## 2 Method

### 2.1 Participants

Cluster sampling was employed to recruit participants from four universities in the northwest region of China. Clusters were defined at the level of universities and classes. Specifically, several classes within each university were selected as sampling units, and all students present in these classes at the time of data collection were invited to participate. A total of 1,327 questionnaires were distributed. Questionnaires were excluded if they failed attention-check items, indicating inattentive responding, or if completion times were unrealistically short, suggesting insufficient engagement with the survey. After data screening, the final sample comprised 1,184 valid questionnaires, yielding an effective response rate of 89.2%. The final sample included 560 males (47.3%) and 624 females (52.7%); 688 freshmen (58.1%), 253 sophomores (21.4%), 152 juniors (12.8%), and 91 seniors (7.7%); 344 only children (29.1%) and 840 non-only children (70.9%); 496 urban students (41.9%) and 688 rural students (58.1%). The study was approved by the ethics committee of the authors’ institution, and informed consent was obtained from all participants prior to data collection.

### 2.2 Measures

#### 2.2.1 Young Schema Questionnaire (Short Form)

Early maladaptive schemas were assessed using the Young Schema Questionnaire - Short Form (YSQ-SF), which was designed by Young and adapted into a Chinese version by Li-Xia et al. (2012). The scale consists of 75 items that are assessed on a 6-point Likert scale from 1 (completely untrue) to 6 (completely true), with higher scores indicating a higher level of maladaptive schemas. The questionnaire includes five subscales: disconnection and rejection, impaired autonomy and performance, impaired limits, other-directedness, and over vigilance and inhibition, with no reverse-coded items. The Cronbach’s *α* for the subscales ranged from 0.70 to 0.92. Confirmatory factor analysis (CFA) demonstrated a good model fit: *χ*^2^/df = 5.612, GFI = 0.949, TLI = 0.966, CFI = 0.974, RMSEA = 0.062, indicating good validity.

#### 2.2.2 Avoidance and Fusion Questionnaire (Short Form)

Psychological flexibility was measured using the Avoidance and Fusion Questionnaire for Youth (AFQ-Y8, Short Form), developed by Greco et al. and adapted into Chinese by Chen et al. (2019). The scale has 8 items and measures a single-factor structure, with no reverse-coded items. Participants rate each item on a 5-point Likert scale ranging from 1 (completely untrue) to 5 (completely true), where higher scores indicate lower psychological flexibility and greater cognitive rigidity. The Cronbach’s *α* for this scale was 0.78. CFA results indicated good model fit: *χ*^2^/df = 2.359, GFI = 0.995, TLI = 0.958, CFI = 0.978, RMSEA = 0.034, confirming good validity. It should be noted that this scale assesses psychological inflexibility; thus, higher scores indicate lower psychological flexibility. Accordingly, interpretations of psychological flexibility in this study are based on this inverse relationship.

#### 2.2.3 Self-Compassion Scale

Self-compassion was assessed using the Self-Compassion Scale (SCS), originally developed by Neff and adapted into Chinese by Gong et al. (2014). The scale consists of 12 items, measuring three dimensions: self-kindness, common humanity, and mindfulness. Each item is rated on a 5-point Likert scale ranging from 1 (almost never) to 5 (almost always), with higher scores indicating greater self-compassion. Items 2, 4, 5, 8, and 11 are reverse-scored. The Cronbach’s *α* for the scale was 0.77. CFA results demonstrated good validity: *χ*^2^/df = 4.052, GFI = 0.983, TLI = 0.903, CFI = 0.935, RMSEA = 0.051.

#### 2.2.4 Perceived Social Support Scale

Perceived social support was measured using the Perceived Social Support Scale (PSSS), developed by Zimet et al. (1988) and adapted into Chinese. The scale consists of 12 items, rated on a 7-point Likert scale from 1 (strongly disagree) to 7 (strongly agree), with higher scores indicating greater perceived social support. The scale includes three subscales, assessing support from family, friends, and other social sources. The Cronbach’s *α* for this scale was 0.896. CFA results demonstrated good model fit: *χ*^2^/df = 3.520, GFI = 0.975, TLI = 0.929, CFI = 0.945, RMSEA = 0.046, indicating high reliability and validity.

### 2.3 Procedure

Prior to data collection, informed consent was obtained from both the participating institutions and all participants. Trained research personnel provided standardized instructions for completing the questionnaires. Participants completed the questionnaires independently in a classroom setting, and responses were collected on-site after completion. To minimize procedural bias, data collection was conducted across the four universities within a similar time frame, and standardized administration procedures were applied to reduce the influence of extraneous variables such as differences in administration and testing conditions.

To ensure data quality, attention-check items were embedded within the questionnaire to identify inattentive responses. In addition, response-time criteria were applied to exclude questionnaires completed in an unrealistically short duration, which may indicate insufficient engagement. These procedures are commonly used in survey-based research to enhance data reliability (Huang et al., 2012).

### 2.4 Data analysis

Data were processed using SPSS 27.0, including descriptive statistics, common method bias tests, and correlation analysis. AMOS 24.0 was used to test mediation and moderation effects.

## 3 Results

### 3.1 Common method bias analysis

As the data were collected using self-report questionnaires, common method bias may be a concern. Therefore, Harman’s single-factor test was conducted using exploratory factor analysis (EFA). The results showed that multiple factors had eigenvalues greater than 1, and the first factor accounted for 26.08% of the total variance, which is below the commonly used threshold of 40%. These findings suggest that common method bias is unlikely to be a serious concern in the present study.

### 3.2 Descriptive statistics and correlation analysis

Table 1 lists the descriptive statistics and correlation matrices of all the variables, including the mean and standard deviation. The results revealed significant relationships between EMSs, perceived social support, self-compassion, and psychological flexibility.

**Table 1:** Correlations and descriptive statistics of study variables.

Variables	M	SD	1	2	3	4
1. EMSs	2.8035	0.8571	1			
2. Perceived social Support	4.9349	0.8374	−0.422**	1		
3. Self-compassion	3.3651	0.5471	−0.34**	0.369**	1	
4. Psychological inflexibility	2.7361	0.6345	0.326**	−0.189**	−0.433**	1

*N* = 1,184, each variable in the model is standardized; Higher scores on the psychological inflexibility measure indicate greater psychological rigidity, reflecting lower levels of psychological flexibility; *p < 0.05, **p < 0.01, ***p < 0.001. The same below.

### 3.3 Mediating role of self-compassion

**Figure 2:**
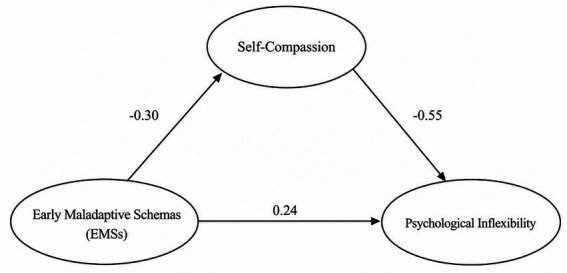
The mediation model.

Mediation analysis was conducted using AMOS 24.0, with the maximum likelihood (ML) estimation method. The Bootstrap method was used for 2,000 resampling iterations, and a bias-corrected 95% confidence interval was applied to test the mediation effect. Model fit was evaluated based on the criteria proposed by Hu and Bentler (1999). In the latent variable mediation model, EMSs were treated as the independent variable, psychological flexibility as the dependent variable, and self-compassion as the mediator. Before constructing the structural model, confirmatory factor analysis (CFA) was performed to ensure good measurement model fit, and item parceling was applied to optimize model estimation. The model fit indices for the mediation model were *χ*^2^/df = 8.842, GFI = 0.926, TLI = 0.897, CFI = 0.916, and RMSEA = 0.081, indicating a good model fit. As shown in Figure 2, EMSs were negatively associated with self-compassion (*β* = −0.303, *p* < 0.001), self-compassion was negatively associated with psychological inflexibility (*β* = −0.548, *p* < 0.001), and EMSs were positively associated with psychological inflexibility (*β* = 0.245, *p* < 0.001), indicating that the direct association was significant.

Further mediation effect testing results are presented in Table 2. The indirect effect was 0.166, with a 95% confidence interval of [0.075, 0.287], which did not contain zero, indicating that the mediation effect was significant. This suggests that self-compassion plays a partial mediating role in the relationship between EMSs and psychological flexibility, with the mediation effect accounting for 40.39% of the total effect.

**Table 2:** Mediation effect model.

Effect type	Effect size	95%CI	*p*-value	Effect ratio
Indirect effect	0.166	[0.075, 0.287]	0.002	40.39%
Total effects	0.411	[0.329, 0.488]	0.001	100%

### 3.4 Moderating role of perceived social support

To improve model estimation and reduce measurement error, item parceling was applied in the construction of latent variables (Little et al., 2002). Item parceling is commonly recommended in structural equation modeling when dealing with complex models, as it can enhance indicator reliability and stabilize parameter estimation, while maintaining acceptable construct validity (Bandalos, 2002). Given that both the independent variable and the moderating variable were modeled as latent constructs, a latent interaction approach was employed to more accurately capture their interaction effect, which cannot be adequately represented using observed variables alone (Marsh et al., 2004).

To further examine the role of perceived social support as a moderating variable, a moderated mediation model was specified to test its moderating effect on the association between EMSs and self-compassion. Prior to analysis, all variables were standardized. Given that the moderating variable was modeled as a latent construct, an interaction term between the independent variable and the moderator was created. Following the recommendations of Wen and Wu (2010), the matched indicator approach was adopted, whereby indicators with high factor loadings were paired with those with similarly high loadings, and indicators with low factor loadings were paired accordingly, to construct the latent interaction term.

**Figure 3:**
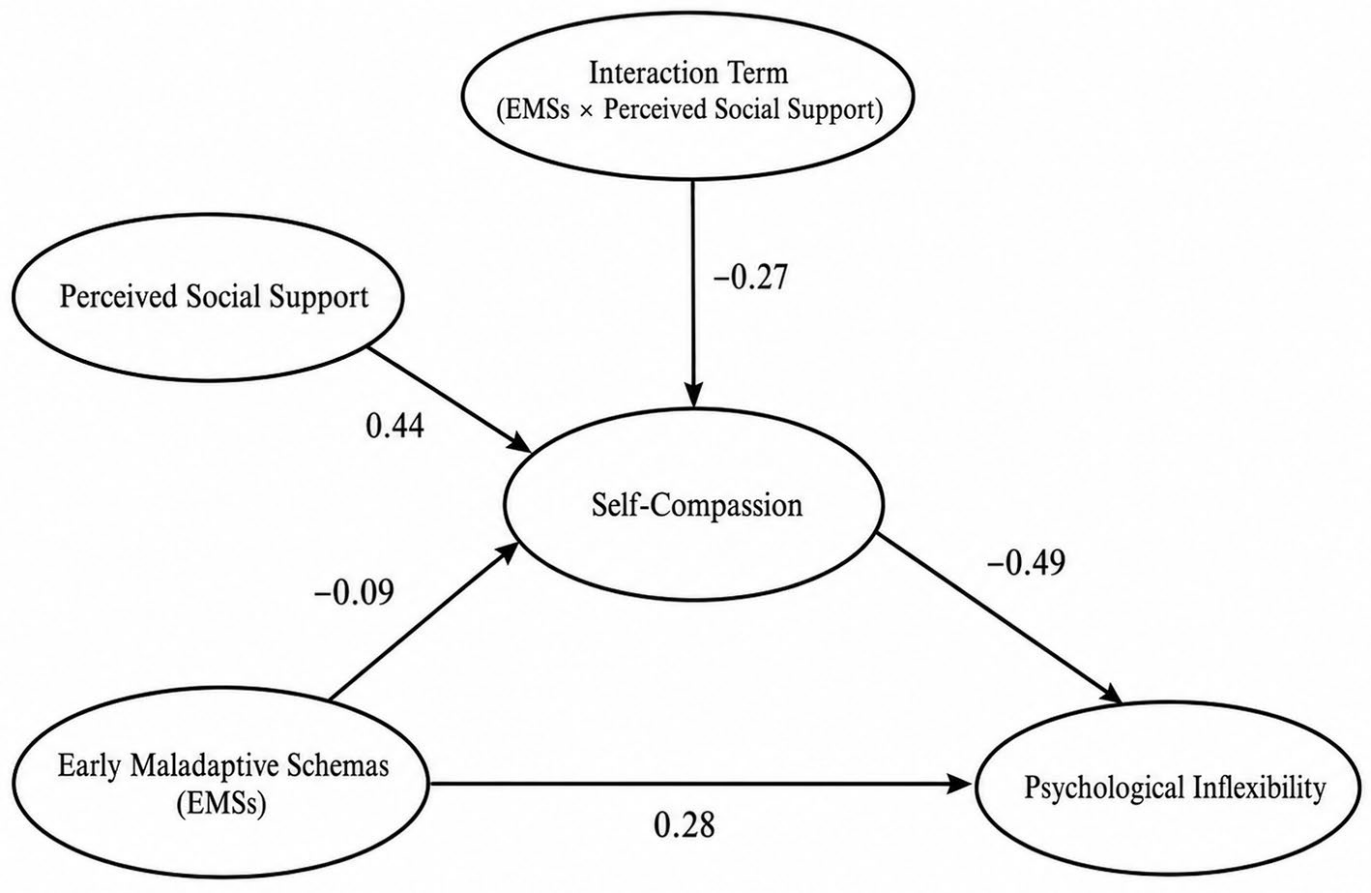
Moderated mediation model.

As shown in Figure 3, the interaction between EMSs and perceived social support was significantly associated with self-compassion (*β* = −0.269, *p* < 0.001), indicating a significant moderating effect. Specifically, the negative association between EMSs and self-compassion was stronger at lower levels of perceived social support and weaker at higher levels, suggesting a buffering role of perceived social support. These results provide support for the proposed moderated mediation model.

## 4 Discussion

### 4.1 EMSs and psychological flexibility

The Avoidance and Fusion Questionnaire was employed in this study, with higher scores indicating greater psychological inflexibility (Chen et al., 2019). The results indicated that EMSs were significantly associated with psychological inflexibility among college students, thereby supporting Hypothesis 1.

This finding is consistent with previous research suggesting that EMSs are linked to reduced psychological flexibility through their influence on cognitive processes (Leahy et al., 2012). Specifically, EMSs are characterized by rigid and negative belief systems, which are associated with experiential avoidance and habitual behavioral patterns, potentially limiting individuals’ capacity to respond flexibly to changing situations (Hosseinzadeh et al., 2021).

From the perspective of ACT, psychological inflexibility is closely related to processes such as cognitive fusion and experiential avoidance (Mohammadkhani et al., 2022). EMSs may be associated with greater reliance on these maladaptive strategies when coping with negative emotions, which may, in turn, be related to reduced emotional acceptance—an essential component of psychological flexibility (Silberstein et al., 2012). In addition, certain schema characteristics may be associated with patterns of rigid evaluation and performance expectations, which are conceptually linked to reduced behavioral flexibility in dynamic contexts (Faraji et al., 2022). Moreover, considering the developmental characteristics of college students, who are often confronted with academic, interpersonal, and career-related challenges, these associations may be particularly salient during this life stage (Sullivan, 2016).

Given these findings, it may be beneficial for university mental health programs to incorporate assessments of EMSs into routine psychological evaluations. Interventions grounded in ACT may be associated with improvements in psychological flexibility, potentially enhancing students’ resilience and social adaptability.

### 4.2 Mediating role of self-compassion

The mediation analysis indicated that self-compassion mediated the association between EMSs and psychological flexibility, supporting Hypothesis 2. Specifically, EMSs were negatively associated with self-compassion, whereas self-compassion was negatively associated with psychological inflexibility. This pattern suggests that higher levels of self-compassion are linked to lower cognitive rigidity and experiential avoidance, and consequently greater psychological flexibility.

This finding can be interpreted within the frameworks of Self-Determination Theory and ACT. According to Self-Determination Theory, autonomy, competence, and relatedness are fundamental to psychological wellbeing (Deci and Ryan, 2012). EMSs are characterized by negative self-evaluations and feelings of helplessness, which have been associated with self-criticism, emotional avoidance, and maladaptive coping strategies (Thimm, 2024). In contrast, self-compassion is associated with responding to distress with acceptance and understanding, and with reduced self-criticism and more adaptive forms of emotional regulation.

From the ACT perspective, psychological flexibility involves openness to internal experiences and engagement in value-based actions (Harris, 2019). Self-compassion may be associated with lower levels of cognitive fusion and experiential avoidance—two core processes underlying psychological inflexibility—thereby relating to greater psychological flexibility. In this sense, the negative association between self-compassion and psychological inflexibility may reflect the role of self-compassion in buffering maladaptive cognitive and emotional processes.

These findings have potential implications for interventions. Programs aimed at enhancing self-compassion, such as those based on ACT and Dialectical Behavior Therapy (DBT), incorporating mindfulness and emotion regulation training, may be associated with improvements in psychological flexibility. Additionally, engagement in reflective and supportive activities, such as reading, meditation, and interpersonal communication, may be related to higher levels of self-compassion and psychological wellbeing among college students.

### 4.3 Moderating role of perceived social support

The moderation analysis supported Hypothesis 3, indicating that perceived social support moderated the association between EMSs and self-compassion. Specifically, the negative association between EMSs and self-compassion was stronger at lower levels of perceived social support and weaker at higher levels, suggesting a buffering role of perceived social support.

This pattern can be interpreted within the framework of attachment theory. Early insecure attachment experiences have been linked to the development of EMSs (Simard et al., 2011). Perceived social support and self-compassion may be interrelated processes, as individuals with more secure attachment tendencies are more likely to perceive available social support and respond to themselves with greater self-compassion when facing challenges (Toplu-Demirtaş et al., 2018). In contrast, individuals with insecure attachment tendencies may be less likely to rely on others for support and may experience greater difficulty in responding to personal failures with self-compassion (Brophy et al., 2020).

In this context, higher levels of perceived social support may be associated with a reduced strength of the negative association between EMSs and self-compassion. One possible explanation is that perceived social support may provide a sense of emotional security and interpersonal connectedness, which may be related to fewer schema-related activations under stress. From this perspective, perceived social support may function as a compensatory interpersonal resource that is associated with more adaptive emotional processing and self-evaluation.

These findings highlight the potential importance of social support in the psychological adjustment of college students. Interventions aimed at strengthening perceived social support, such as peer support programs and enhanced teacher–student interactions, may be associated with higher levels of self-compassion and more adaptive psychological functioning.

In summary, the present findings indicate that EMSs are associated with psychological flexibility both directly and indirectly through self-compassion, while perceived social support may exert a buffering role in this process. These results underscore the relevance of both intrapersonal and interpersonal factors in understanding psychological flexibility. However, given the cross-sectional design, the findings should be interpreted with caution, and causal inferences cannot be drawn.

## 5 Conclusions and implications

In conclusion, the present study provides evidence that EMSs are associated with psychological flexibility among college students, both directly and indirectly through self-compassion. In addition, perceived social support appears to moderate the association between EMSs and self-compassion.

From a theoretical perspective, the results are broadly consistent with frameworks such as ACT and attachment theory, underscoring the relevance of cognitive–emotional processes and relational contexts in shaping adaptive functioning.

From an applied perspective, the findings suggest that interventions targeting EMSs, self-compassion, and perceived social support may be relevant for promoting psychological adjustment among college students. In particular, approaches grounded in cognitive-behavioral and mindfulness-based frameworks may be associated with improvements in emotion regulation and coping processes. Likewise, efforts to enhance perceived social support may be linked to more adaptive psychological outcomes.

Nevertheless, these conclusions should be interpreted in light of the study’s cross-sectional design, which limits causal inference. Future research employing longitudinal and experimental designs is warranted to further clarify the directionality and underlying mechanisms of these associations.

## 6 Study limitations

The present study has several limitations that should be acknowledged. First, the sample was drawn from four universities in the Northwest region of China, which may limit the generalizability of the findings to other populations or age groups. Future research could include more diverse samples across different demographic and cultural contexts to enhance external validity. Second, the cross-sectional design precludes causal inference and limits conclusions regarding the directionality of the observed associations. Future studies employing longitudinal or experimental designs are warranted to further examine the temporal relationships and underlying mechanisms among EMSs, self-compassion, perceived social support, and psychological flexibility. Third, although the present study focused on key psychological and interpersonal variables, broader contextual factors, such as social and cultural influences, were not fully considered. Future research may benefit from incorporating these factors to provide a more comprehensive understanding of the processes underlying psychological flexibility.

## Data Availability

The raw data supporting the conclusions of this article will be made available by the authors, without undue reservation.
